# Japanese single-center experience of abdominal aortic aneurysm repair over 20 years: should open or endovascular aneurysm repair be performed first?

**DOI:** 10.1007/s00595-023-02663-3

**Published:** 2023-03-24

**Authors:** Katsuhiro Yamanaka, Mari Hamaguchi, Shunya Chomei, Taishi Inoue, Atsunori Kono, Takanori Tsujimoto, Yojiro Koda, Hidekazu Nakai, Atsushi Omura, Takeshi Inoue, Masato Yamaguchi, Koji Sugimoto, Kenji Okada

**Affiliations:** 1 Division of Cardiovascular Surgery, The Department of Surgery, University of Kobe, Graduate School of Medicine, 7-5-2 Kusunoki-Cho, Chuo-Ku, Kobe, 650-0017 Japan; 2 The Department of Radiology, University of Kobe, Kobe, Japan

**Keywords:** Abdominal aortic aneurysm, Open repair, Endovascular aneurysm repair, Instruction for use

## Abstract

**Purpose:**

The present study analyzed the outcomes of our experience with abdominal aortic aneurysm (AAA) repair over 20 years using endovascular aortic repair (EVAR) with commercially available devices or open aortic repair (OAR) and reviewed our surgical strategy for AAA.

**Methods:**

From 1999 to 2019, 1077 patients (659 OAR, 418 EVAR) underwent AAA repair. The OAR and EVAR groups were compared retrospectively, and a propensity matching analysis was performed.

**Results:**

EVAR was first introduced in 2008. Our strategy was changed to an EVAR-first strategy in 2010. Beginning in 2018, this EVAR-first strategy was changed to an OAR-first strategy. After propensity matching, the overall survival in the OAR group was significantly better than that in the EVAR group at 10 years (p = 0.006). Two late deaths due to AAA rupture were identified in the EVAR group, although there were no significant differences between the OAR and EVAR groups with regard to the freedom from AAA-related death at 10 years. The rate of freedom from aortic events at 10 years was significantly higher in the OAR group than in the EVAR group (p < 0.0001).

**Conclusion:**

The rates of freedom from AAA-related death in both the OAR and EVAR groups were favorable, and the rate of freedom from aortic events was significantly lower in the EVAR group than in the OAR group. Close long-term follow-up after EVAR is mandatory.

## Introduction

Abdominal aortic aneurysm (AAA) is a common yet inconspicuous disease that can lead to devastating complications. One such complication is AAA rupture, which has reported mortality rates of up to 90% [1]. Three major randomized controlled trials demonstrated the superiority of EVAR to open aortic repair (OAR) in preventing 30-day mortality [2–4]. That being said, EVAR and OAR result in similar long-term survival rates [8–10]. Furthermore, EVAR has been associated with increased rates of re-intervention [5, 6, 8, 9]. However, in many institutions, EVAR is performed as the first-line procedure.

The surgical outcomes of AAA in Asia cannot be simply compared with those in previous reports in Western populations due to differences in patient characteristics between these two populations, such as in the body mass index and body weight [10, 11]. Therefore, it is very difficult to determine whether OAR or EVAR is more appropriate for managing AAA repair in an Asian population.

The present study therefore analyzed the outcomes of EVAR and OAR at a single Japanese center over a 20-year period and reviewed our surgical strategy for AAA.

## Study design and patient selection

This single-center, retrospective study was approved by the Institutional Review Board (IRB) of the Kobe University School of Medicine (IRB number: #B220178). All consecutive patients with infrarenal AAA undergoing EVAR or OAR between October 1999 and May 2019 were included in this study. The indication for treatment was an AAA diameter ≥ 50 mm or a diameter expansion ≥ 5 mm over a 6-month period. Ruptured AAA was defined as symptomatic AAA with an unstable circulatory condition. Cases of contained rupture, which refers to symptomatic AAA without hemodynamic collapse, were excluded from the ruptured AAA group. Infected abdominal aortic aneurysms were also excluded in this cohort, although infection after OAR or EVAR was recognized as a complication.

Our surgical strategy for AAA changed over time. EVAR was introduced at our institution in 2008, and in 2010, an EVAR-first strategy was implemented. Beginning in 2018, however, this EVAR-first strategy was changed to an OAR-first strategy. We devised a patient-centered approach, choosing the technique based on individual patient characteristics. Shaggy aorta or disseminated intravascular coagulation (DIC) was basically a contraindication for EVAR. The eligibility criteria of instructions for use (IFU) are a short proximal neck (≥ 15 mm), severe neck angulation (≤ 60°), poor access (iliac artery diameter ≥ 7.5 mm), and short distal landing zone (≥ 15 mm). When all criteria were present, the patient was assigned to the IFU group. If even one of these criteria was absent, the patient was assigned to the non-IFU group.

### Surgical techniques of OAR

OAR was performed using a midline incision and transperitoneal approach or a pararectal incision and retroperitoneal approach, depending on the surgeon. The AAA was replaced just below the renal arteries with an infrarenal or suprarenal aortic clamp using a straight or bifurcated graft.

### Surgical techniques of EVAR

EVAR was also carried out by our professional interventional team consisting of cardiovascular surgeons and interventional radiologists. The procedure was performed with bilateral groin cutdowns using one of the following stent grafts: Endurant (Medtronic, Minneapolis, MN, USA), Excluder (Gore, Flagstaff, AZ, USA), Zenith (Cook Medical, Bloomington, IN, USA), Powerlink (Endologix, Irvine, CA, USA), AFX (Endologix), or Aorfix (Lombard Medical, Oxon, UK). We divided the time periods (2008–2019) into three terms: early term (2008–2011), middle term (2012–2017), and late term (2018-present) based on the EVAR strategy. In the middle term, preemptive inferior mesenteric artery (IMA) embolization with EVAR was performed to prevent type II endoleak [12]. In the late term, preemptive embolization of the lumbar arteries was done in addition to that of the IMA. Technical success was defined as complete delivery of a stent graft and no type I or III endoleak at completion angiography.

### Definition of aortic events and AAA-related death

“Aortic events” in the OAR group were defined as hospital death, postoperative complications requiring surgical repair, or ileus requiring placement of a nasointestinal ileus tube or long tube for gastrointestinal decompression. “Aortic events” in the EVAR group were defined as hospital death, open conversion, or re-intervention. “AAA-related death” was defined as death caused by AAA.

### Statistical analyses

Statistical analyses were performed using the JMP software program for Macintosh, version 15.0.0 (SAS Institute, Cary, NC, USA). All continuous data are expressed as the mean ± standard error. The comparison of clinical characteristics was done with the χ^2^ test for categorical variables and nonpaired *t*-tests for continuous variables. The risk factors for aortic events were evaluated with cox proportional hazard analyses. The rates of late survival, freedom from AAA-related death, freedom from aortic events, and freedom from re-intervention were analyzed using the Kaplan–Meier method. A p-value < 0.05 was considered significant for all statistical comparisons.

**Fig. 1:**
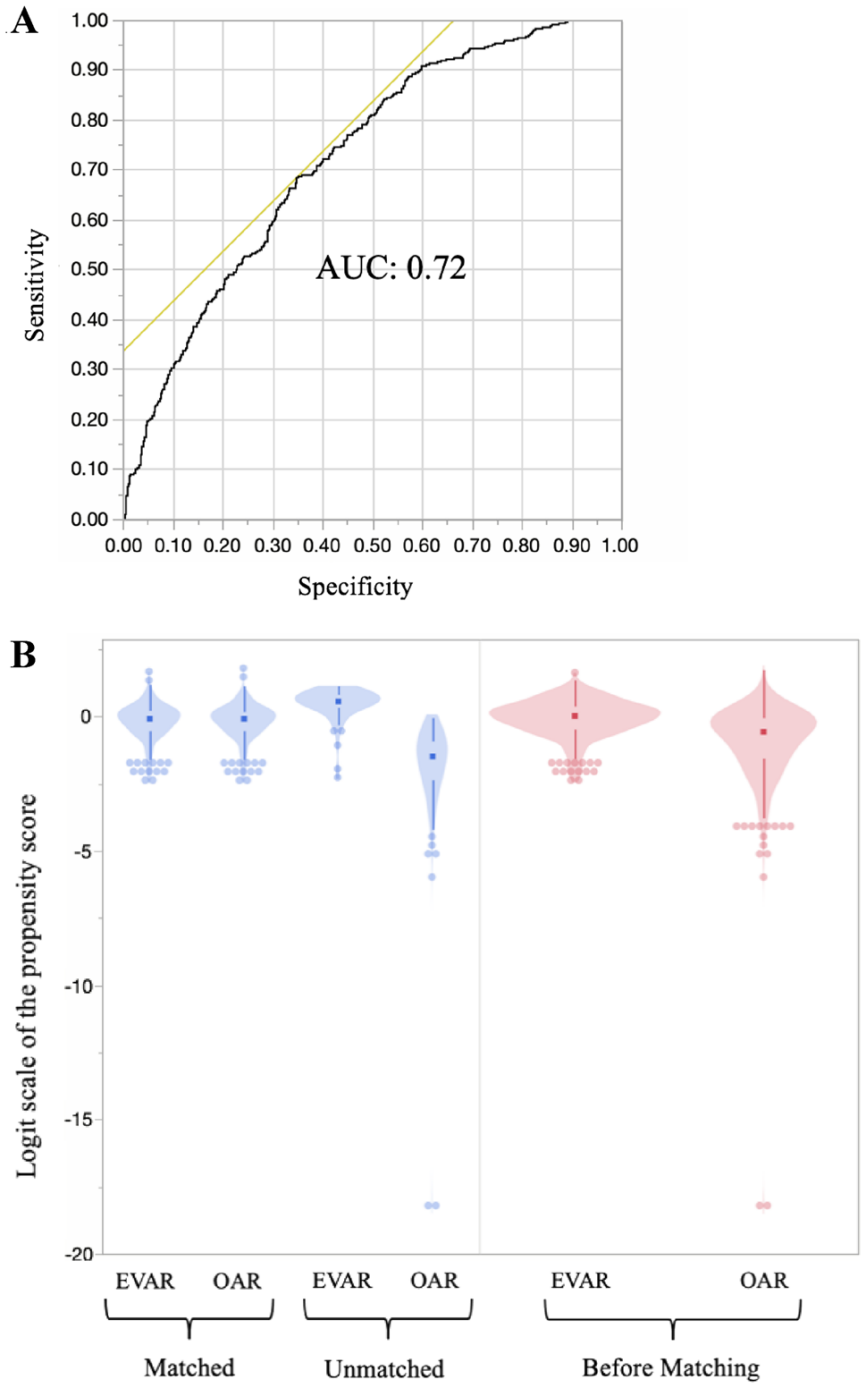
**A** Receiver operating characteristic (ROC) curve for the propensity score-matched analysis. The area under the ROC curve (AUC) was 0.72. **B** Violin plots of the matched and unmatched groups as well as the groups before matching showed the distribution of dots based on the logit scale of the propensity score. The distribution of dots in the matched OAR groups was very similar to that of the matched EVAR groups

Propensity score matching was performed to reduce the potential bias in baseline characteristics between the OAR and EVAR groups (Fig. 1A, B). The propensity score was obtained using a logistic regression algorithm. The covariates for propensity score calculation included the preoperative variables presented in Table 1. Patients were matched using 1:1 nearest neighbor matching with a caliper size of 0.2. A total of 322 propensity score-matched pairs were ultimately identified for the final analysis. Standardized mean differences were compared for all covariates after matching.

**Table 1:** Preoperative patients’ characteristics

Variables	Total cohort (n = 1077)				Propensity-matched cohort (n = 644)			
	OAR (659 cases)	EVAR (418 cases)	SMD	P value	OAR (322cases)	EVAR (322 cases)	SMD	P value
Age	73.6 ± 0.3	76.7 ± 0.4	− 0.565	< 0.0001	75.3 ± 0.3	75.5 ± 0.3	− 0.666	0.75
Male	522 (79.2%)	401 (95.9%)	− 0.522	< 0.0001	314 (97.5%)	322(100%)	− 0.226	0.007
BMI	23.0 ± 0.1	23.0 ± 0.2	− 0.076	0.872	23.1 ± 0.2	23.1 ± 0.2	0.000	0.88
Rupture	101 (15.3%)	8 (1.9%)	0.492	< 0.0001	11 (3.4%)	5 (1.5%)	0.123	0.20
Hypertension	468 (76.3%)	319 (76.3%)	0	0.991	241 (74.8%)	250 (77.6%)	− 0.065	0.45
Dyslipidemia	242 (39.5%)	181 (43.3%)	− 0.077	0.295	131 (40.6%)	145 (45.0%)	− 0.089	0.30
CKD (Cre > 1.5)	101 (16.4%)	64 (15.4%)	0.027	0.675	44 (13.6%)	45 (13.9%)	0.000	1.00
COPD	75 (12.2%)	63 (15.0)	− 0.081	0.194	44 (13.6%)	49 (15.2)	− 0.008	0.65
DM	85 (13.8%)	56 (13.4%)	0.011	0.579	43 (13.3%)	48 (14.9%)	− 0.045	0.65
CVD	80 (13.0%)	44 (10.5%)	0.077	0.214	39 (12.1%)	38 (11.8%)	0.009	1.00
Smoking	378 (61.8%)	308 (73.6%)	− 0.254	< 0.0001	245 (76.0%)	245 (76.0%)	0.000	1.00
PCI	55 (8.9%)	32 (7.6%)	0.047	0.457	22 (6.8%)	24 (7.4%)	− 0.023	0.87
CABG	15 (2.4%)	15 (3.5%)	− 0.065	0.284	10 (3.1%)	12(3.7%)	− 0.033	0.82
Aortic surgery	55 (8.9%)	9 (2.1%)	0.301	< 0.0001	9 (2.8%)	9 (2.8%)	0.301	1.00

*OAR* open aortic repair, *EVAR* endovascular aortic repair**,** *BMI* body mass index, *CKD* chronic kidney disease, *COPD* chronic obstructive pulmonary disease, *DM* diabetes mellitus, *CVD* cerebrovascular disease, *PCI* percutaneous coronary intervention, *CABG* coronary artery bypass grafting, *SMD* standardized mean difference

## Results

### Population characteristics

**Fig. 2:**
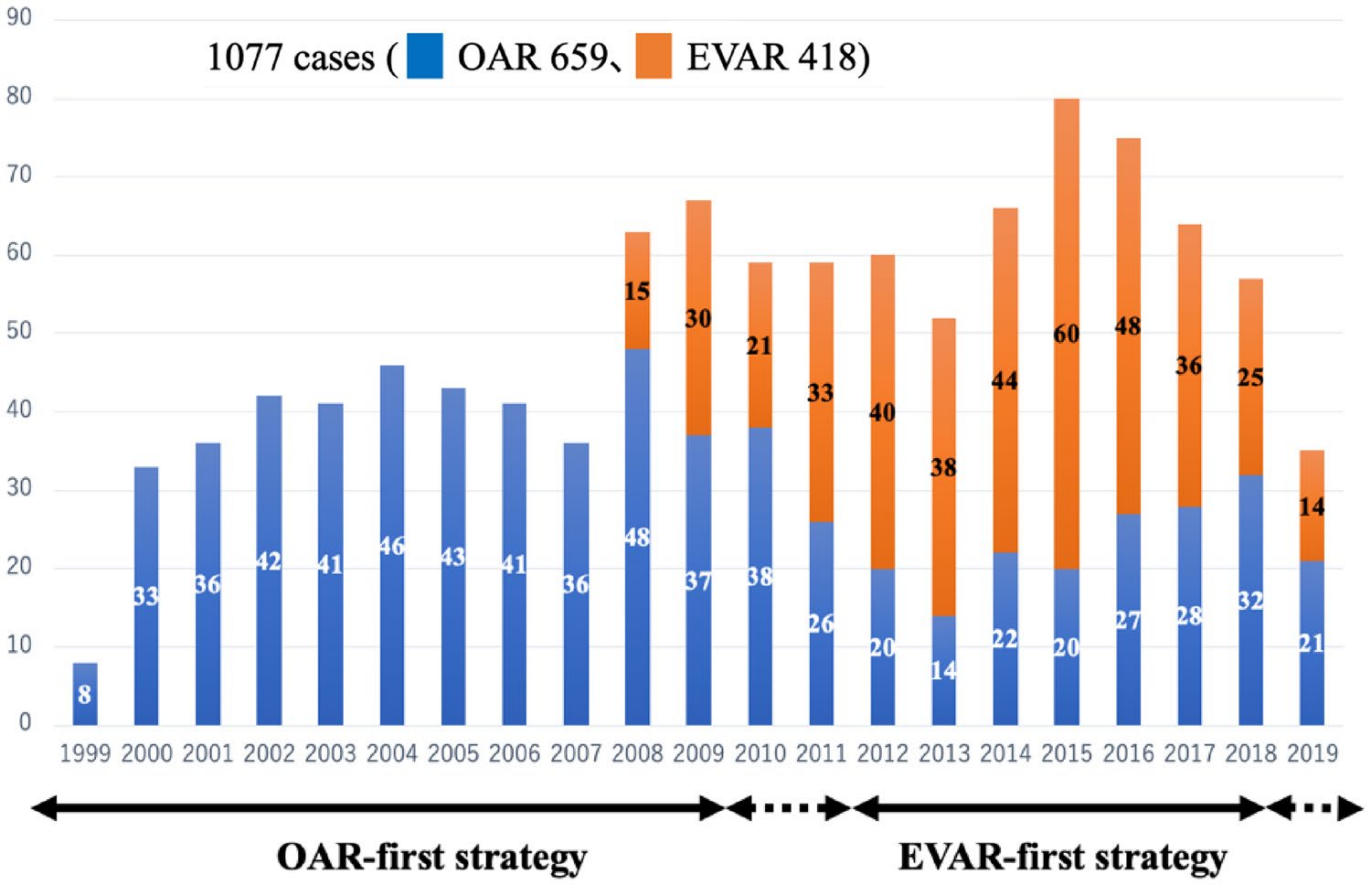
Number of AAA cases. AAA was treated with OAR first strategy from 1999 to 2009. EVAR was introduced in 2008, and OAR first strategy was changed to EVAR first strategy in 2012. After 2018, the EVAR first strategy was changed to the OAR first strategy based on the characteristics of each individual patient

A total of 1077 patients underwent AAA repair between October 1999 and May 2019. Of these, 659 patients (61%) underwent OAR, and 418 patients (39%) underwent EVAR. Figure 2 shows the number of each operation performed annually. The median follow-up of the OAR group was 3.16 (0.13–18.1) years, and that of the EVAR group was 3.09 (0.02–11.2) years. Clinical characteristics in this study are shown in Table 1. The mean age in the EVAR group (76.7 ± 0.4 years old) was significantly higher than that in the OAR group (73.6 ± 0.3 years old) (p < 0.0001). The OAR group also had significantly more patients with ruptured AAA than the EVAR group (OAR, 15.3%; EVAR, 1.9%; p < 0.0001). Regarding the 322 propensity score-matched pairs, the EVAR group had significantly more male patients than the OAR group (p = 0.007).

Regarding the stent grafts we used, an Endurant was used in 173 patients (41%), Excluder in 129 (31%), Zenith in 72 patients (17%), Powerlink in 29 (7%), AFX in 9 (2%), and Aorfix in 7 (2%). Within the EVAR group, the IFU group comprised 352 patients (86.0%) and the non-IFU group 66 patients (14.0%). Of the 66 non-IFU patients, a short proximal neck was observed in 5, severe neck angulation in 25, poor access in 2, reversed taper in 12, and short distal landing zone in 22. The mean diameter (maximal minor axis) of AAA significantly decreased from 51.5 ± 0.5 mm (preoperative) to 47.8 ± 0.5 mm (postoperative) (p < 0.001). Enlargement of the AAA compared with the preoperative diameter (> 5 mm) was observed in 66 patients (14.8%), whereas 160 patients (40.4%) had sac regression (> 5 mm). Technical success was achieved in 398 patients (95.2%; Type Ia, n = 17; Type III, n = 3).

### Mortality

The 30-day mortality rate was 0.72% (n = 3) in the EVAR group and 2.28% (n = 10) in the OAR group (p = 0.24). The causes of 30-day death were as follows: aortic dissection caused by balloon expansion in 1 (EVAR), aspiration pneumonia in 2 (EVAR), and multiple organ failure (MOF) in 10 (OAR). After the matching analysis, the 30-day mortality rate was 0.93% (n = 3) in the EVAR group and 0.62% (n = 2) in the OAR group (p = 0.65). The hospital mortality rate in the OAR group (3.19%, n = 20) was significantly higher than in the EVAR grope (0.96%, n = 4) (p = 0.024). Aside from cases of 30-day death, the causes of hospital death were as follows: pancreatitis in 1 patient (EVAR), intestinal necrosis in 2 patients (OAR), pneumonia in 2 patients (OAR), and MOF in 6 patients (OAR). After matching, the hospital mortality rate was 1.24% (n = 4) in the EVAR group and 1.24% (n = 4) in the OAR group (p = 1.00). When we focused on the only elective cases, the 30-day mortality and hospital mortality rates in the OAR group were 0% (n = 0) and 0.60% (n = 4) respectively, whereas those in the EVAR group were 0.48% (n = 2) and 0.72% (n = 3) respectively.

**Fig. 3:**
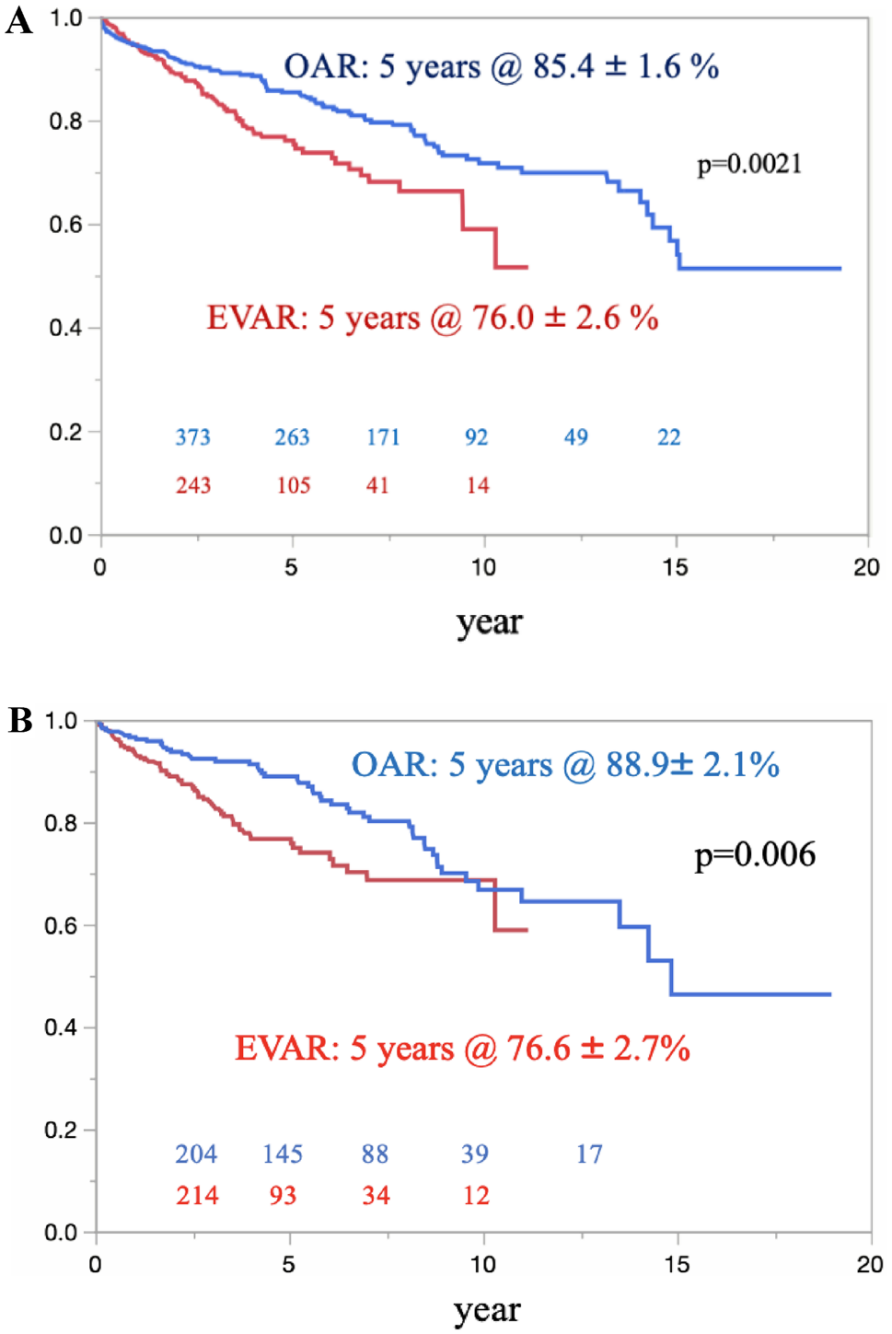
The overall survival was significantly better in the OAR group than in the EVAR group in the total cohort. (**A**) Curves for the overall cohort. (**B**) Curves of the propensity score-matched cohort

**Fig. 4:**
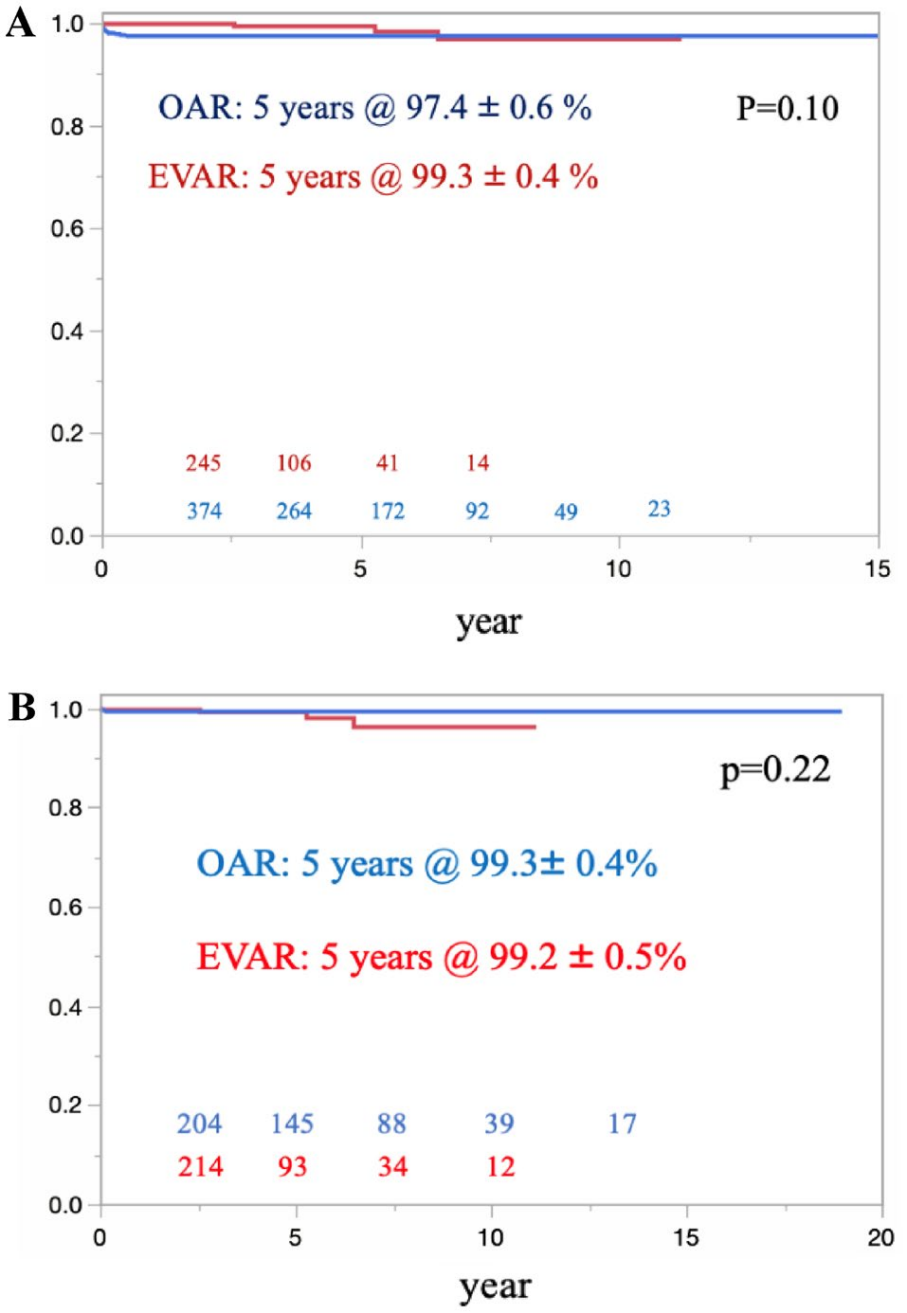
In terms of freedom from AAA-related death, there were no significant differences between the OAR and EVAR groups. (**A**) Curves for the overall cohort. (**B**) Curves of the propensity score-matched cohort

The overall survival in the OAR and EVAR groups was 85.4% ± 1.6% and 76.0% ± 2.6%, respectively, at 5 years (p = 0.0021) (Fig. 3A). Eighty-seven late deaths were confirmed in the OAR group, compared with 95 late deaths in the EVAR group. The causes of late death are shown in Table 2. Regarding freedom from AAA-related late death, there were no significant differences between the 2 groups (p = 0.10) (Fig. 4A). However, four patients with late rupture of AAA were identified in the EVAR group. Of these, two patients died, while the other two survived; one patient underwent OAR, and the other underwent EVAR. There were no cases of late rupture of AAA and no AAA-related late deaths in the OAR group. In the propensity score-matched cohort, the overall survival in the OAR and EVAR groups was 88.9% ± 2.1% and 76.6% ± 2.7%, respectively, at 5 years (p = 0.006) (Fig. 3B). There were no significant differences between the groups in terms of freedom from AAA-related late death (p = 0.10) (Fig. 4B).

**Table 2:** Causes of late death

OAR	87	EVAR	95
Malignancy	18	Malignancy	32
Pneumonia	15	Unknown	28
Unknown	14	Pneumonia	13
ACS	9	CVD	8
CVD	9	HF	7
Rupture of TAA	7	Rupture of TAA	2
RF	5	Rupture of AAA	2
HF	4	RF	2
MOF	2	Sepsis	1
Ileus	1		
Intestinal necrosis	1		
Aortic dissection	1		
liver cirrhosis	1		

*OAR* open aortic repair, *EVAR* endovascular aortic repair**,** *ACS* acute coronary syndrome, *CVD* cerebrovascular disease, *RF* renal failure, *HF* heart failure, *MOF* multiple organ failure, *TAA* thoracic aortic aneurysm, *AAA* abdominal aortic aneurysm

### Aortic events and risk factors for aortic events

**Fig. 5:**
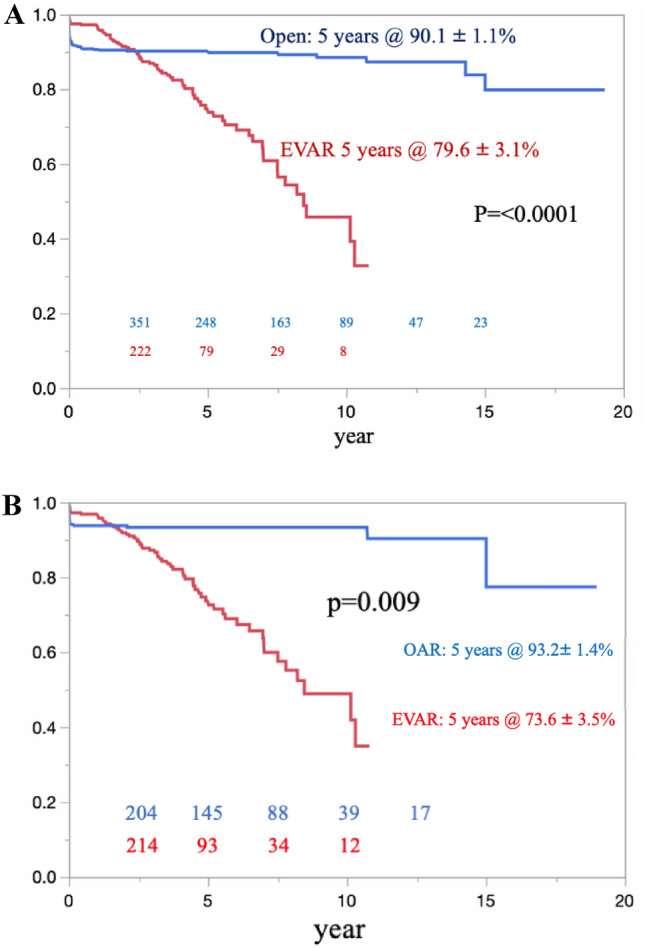
The rate of freedom from aortic events was significantly higher in the OAR group than in the EVAR group. (**A**) Curves for the overall cohort. (**B**) Curves of the propensity score-matched cohort

The rate of freedom from aortic events at 5 years was significantly higher in the OAR group than in the EVAR group (OAR 90.1% ± 1.1%; EVAR 79.6% ± 3.1%) (p < 0.0001) (Fig. 5A). In the propensity score-matched analysis, the rate of freedom from aortic events at 5 years was also significantly higher in the OAR group than in the EVAR group (OAR 93.2% ± 1.4%; EVAR 73.6% ± 3.5%, p = 0.009) (Fig. 5B). Aortic events in each group are shown in Table 3. In the OAR group, gastrointestinal decompression was performed in 27 patients due to ileus, and 21 patients required surgical intervention. In contrast, in the EVAR group, 63 patients required 83 reinterventions, including 24 conversions to open repair (Table 4).

**Table 3:** Aortic events in the OAR and EVAR groups

OAR (n = 659)	N	EVAR (n = 418)	N
Hospital death	11	Hospital death	4
Ileus	27	Re-intervention	63
Intestinal necrosis	11		
Acute arterial occlusion	3		
Aortoduodenal fistula	2		
Distal anastomotic stenosis	2		
Abdominal incisional hernia	2		
Pseudoaneurysm of anastomosis	1		
Total	59		67

*OAR* open aortic repair, *EVAR* endovascular aortic repair

**Table 4:** Re-intervention after EVAR

Cause or procedure	83 re-interventions
Coil embolization or NBCA (Type 2)	32
Cuff (Type 1a)	5
Leg extension (Type 1b)	10
re-EVAR (Type 3 or 4)	7
Leg occlusion	4
Acute arterial occlusion	1
Open conversion	24

*NBCA* N-butyl-2-cyanoacrylate, *EVAR* endovascular aortic repair

**Fig. 6:**
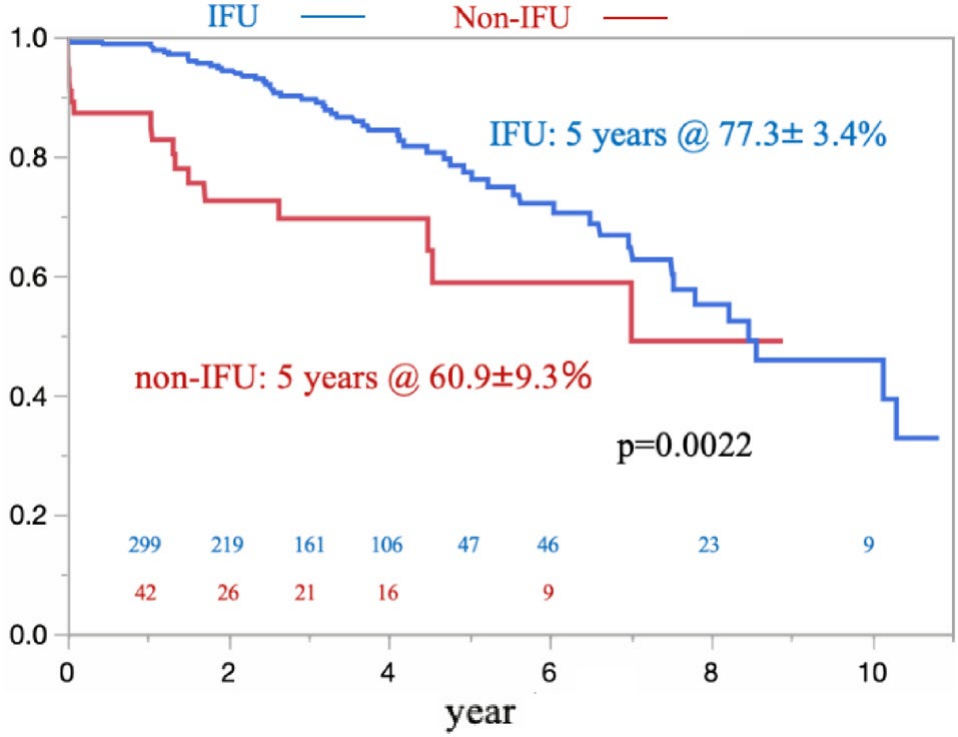
The rate of freedom from re-intervention was significantly higher in the IFU group than in the non-IFU group. However, the advantage inherent to the inside IFU group disappeared about eight years after EVAR

**Fig. 7:**
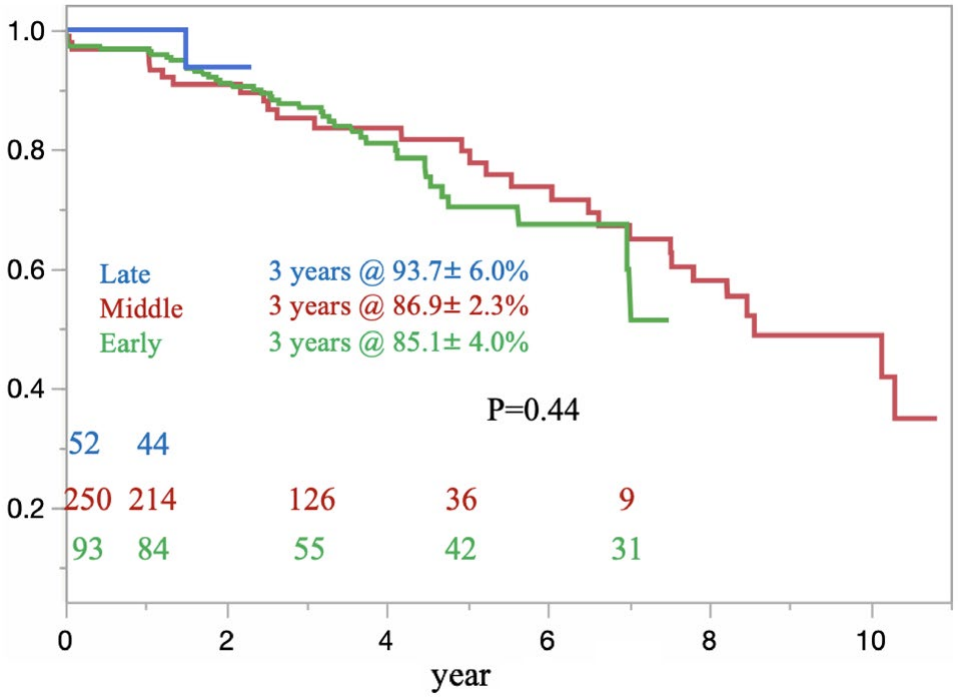
No significant improvement in freedom from re-intervention after EVAR was detected among the early, middle, and late terms

The results of univariate and multivariate analyses for aortic events in the OAR group are shown in Table 5. After the multivariate analysis, only ruptured AAA was associated with aortic events following OAR (OR, 7.27; P < 0.001). Table 6 shows the results of univariate and multivariate analyses for aortic events in the EVAR group. The multivariate analysis showed that type Ia endoleak (OR, 5.57; P = 0.0006) and type II endoleak (OR, 6.07; P < 0.001) were associated with aortic events after EVAR. Regarding the re-intervention after discharge in the EVAR group, the rate of freedom from re-intervention was significantly higher in the IFU group than in the non-IFU group (IFU 77.3% ± 3.4%; non-IFU 60.9% ± 9.3%) (p = 0.0022) (Fig. 6). Furthermore, in the EVAR group, no significant improvements in the rate of freedom from re-intervention were noted among the three terms (early vs. middle vs. late) (Fig. 7).

**Table 5:** Risk factors for an aortic event in the OAR group identified by univariate and multivariate analyses

Factor	Univariate		Multivariate	
	OR (95%CI)	P value	OR (95%CI)	P value
Age ≧ 75	2.17 (1.09–4.33)	0.026	1.54 (0.92–2.58)	0.09
Male	4.71 (1.11–19.9)	0.009	1.59 (0.55–1.70)	0.10
Rupture	6.57 (3.23–13.3)	< 0.001	7.27 (4.41–11.9)	< 0.001
BMI	1.05 (0.98–1.11)	0.105		
HT	1.16 (0.53–2.50)	0.705		
CKD	1.16 (0.53–2.56)	0.696		
CVD	1.39 (0.61–3.17)	0.428		
COPD	1.34 (0.54–3.27)	0.518		
Current smoking	1.30 (0.58–2.94)	0.809		
Past smoking	1.33 (0.62–2.84)	0.454		
Aortic surgery	0.57 (0.17–1.91)	0.367		
PCI	0.27 (0.03–2.02)	0.203		
CABG	1.18 (0.15–8.98)	0.869		

*OR* odds ratio, *CI* confidence interval, *BMI* body mass index, *HT* hypertension, *CKD* chronic kidney disease, *CVD* cerebrovascular disease, *COPD* chronic obstructive pulmonary disease, *PCI* percutaneous coronary intervention, *CABG* coronary artery bypass grafting, *OAR* open aortic repair

**Table 6:** Risk factors for an aortic event in the EVAR group identified by univariate and multivariate analyses

	Univariate		Multivariate	
	OR (95%CI)	P value	OR (95%CI)	P value
Age ≧ 75	1.17 (0.67–2.05)	0.561		
Male	0.37 (0.08–1.67)	0.200		
Inside the IFU	0.46 (0.21–1.03)	0.060		
Pre. AAA size	1.01 (0.97–1.04)	0.526		
Type 1A	4.45 (1.80–10.9)	0.001	5.57 (2.62–12.7)	0.0006
Type 1B	5.28 (1.35–20.6)	0.016	2.22 (0.76–6.44)	0.1760
Type 2	6.44 (3.36–12.3)	< 0.001	6.07 (3.53–10.4)	< 0.001
Type 3	4.08 (1.08–15.4)	0.038	2.38 (0.98–5.74)	0.07
Type 4	1.30 (0.23–7.37)	0.762		
BMI	1.03 (0.95–1.11)	0.379		
Rupture	0.57 (0.7Factor6–21.4)	0.101		
HT	1.68 (0.77–3.65)	0.185		
COPD	0.56 (0.19–1.59)	0.278		
Current smoking	0.83 (0.26–2.59)	0.749		
Past smoking	1.40 (0.67–2.91)	0.357		
DM	0.40 (0.13–1.21)	0.406		
DL	0.48 (0.25–0.92)	0.029	0.64 (0.38–1.08)	0.1446
CKD	1.55 (0.68–3.50)	0.291		
PCI	2.2.9 (0.88–5.90)	0.086		
Antiplatelet or Anticoagulant	2.69 (0.17–41.4)	0.477		

*OR* odds ratio, *CI* confidence interval, *IFU* instructions for use, *AAA* abdominal aortic aneurysm, *BMI* body mass index, *HT* hypertension, *COPD* chronic obstructive pulmonary disease, *DM* diabetes mellitus, *DL* dyslipidemia, *CKD* chronic kidney disease, *PCI* percutaneous coronary intervention, *EVAR* endovascular aortic repair

## Discussion

In the present study, EVAR and OAR resulted in similarly favorable long-term outcomes of freedom from AAA-related death. Despite these findings, however, delayed rupture after EVAR, which was a rare but lethal complication, was identified, whereas there was no delayed rupture after OAR. EVAR led to a significantly increased number of aortic events compared with OAR. Furthermore, patients with non-IFU had a significantly higher rate of re-intervention than those with IFU. This study was unable to demonstrate any significant improvement in freedom from re-intervention in the EVAR group compared with OAR.

In some centers [13–15], EVAR has become the preferred method over OAR because of the reduced invasiveness. At our institution, EVAR was introduced in 2008 and performed as a the first-line treatment option between 2010 and 2017. OAR during this time period was used to treat patients with a more challenging anatomy, such as those with suprarenal clamping, tortuous aorta, or a high degree of atherosclerotic change. Furthermore, OAR was basically performed in patients with shaggy aorta or DIC. Nevertheless, the present study revealed favorable outcomes in the OAR group compared with the EVAR group. Although age was found to be one of the most important factors for determining the surgical strategy, the strategy was not determined based on just the age; indeed, the severity of chronic obstructive pulmonary disease (COPD) and frailty were significant factors for deciding the surgical procedure as well. In addition, other comorbidities and the patient’s history were also considered.

This study showed that the early mortality rate in the EVAR group was favorable and equivalent to that in previous randomized trials (DREAM trial, EVAR-1 trial, and OVER trial) [2–4]. EVAR (0.9%) had a significantly lower hospital mortality than OAR (3.1%) before the matching analysis. In total, 20 hospital deaths after OAR were confirmed. Of these 20 deaths, 16 were found to have involved a ruptured AAA, which we defined in this study as having a hemodynamically unstable condition. A total of 109 patients had a ruptured AAA in the present study. Of those 109 patients, OAR was performed in 101 (92.6%). After excluding these patients from the cohort, the significant differences in hospital mortality disappeared (OAR 0.15%; EVAR 0.23%). Furthermore, a propensity score-matched analysis also showed that the significant differences in hospital mortality vanished.

Conversely, the hospital mortality rate in the OAR group (3.1%) was better than the values reported in the DREAM trial (4.6%), EVAR-1 trial (6.2%), and OVER trial (6.6%) even though the OAR group in this study included many complex patients, such as those with ruptured AAA or with a challenging anatomy [2–4]. In general, cardiac events are well described as the major cause of hospital death after OAR [16]. However, no cardiac events were identified in the present study. In most Japanese institutions, preoperative screening examinations of coronary artery disease and cerebrovascular disease are performed in patients with AAA. This may explain the relatively good outcomes of OAR noted in our study.

Besides screening examinations, obesity has also been linked to significant complications with AAA repair, and there are notable differences between the patients included in our study and those in earlier trials. Giles et al. [17] and Khorgami et al. [18] showed that obesity was associated with an increased risk of mortality after OAR. The mean body mass index (BMI) in the DREAM trial, EVAR-1 trial, and OVER trial was 26.6, 26.4, and 28.7, respectively, while that in the present study was 23.0. In the OVER trial, 10.1% of patients who underwent OAR were obese, with a BMI exceeding 35. A systemic review by Saedon et al. reported that, in obese patients undergoing AAA repair, EVAR was superior to OAR regarding postoperative morbidity and mortality [19]. Some studies have shown that obesity is a risk factor for wound infections and renal failure, although obesity in itself is not an independent risk factor of mortality [20, 21]. Therefore, obesity is directly or indirectly involved in mortality after OAR.

Regarding the long-term outcomes, the overall survival was significantly better in the OAR group than in the EVAR group. This was unsurprising, as EVAR was performed in predominantly elderly and high-risk patients. However, even in the propensity score-matched cohort, the overall survival of the OAR group was significantly better than that of the EVAR group, although the survival in the EVAR group at 10 years was almost equal to that of the OAR group. This is probably because the EVAR group had more patients with malignancy and frailty than the OAR group. Indeed, in the EVAR group, 32 patients (7.6%) died because of malignancy, while 18 (2.7%) in the OAR group died due to malignancy.

EVAR and OAR resulted in similarly favorable rates of freedom from AAA-related death. However, 4 patients (0.9%) with late aneurysm rupture were identified only in the EVAR group (0.8, 1, 4, and 8 years after EVAR), with none found in the OAR group. Two of these four cases of rupture resulted in death, while the other two were successfully treated by emergent re-intervention (re-EVAR in one and OAR in one). The incidence rate of late rupture is reported to be 1.3–7% [7, 22–25]. A Swedish national investigation performed by Andersson et al. demonstrated that the independent risk factors for late rupture were ruptured AAA at initial EVAR and age. They concluded that late rupture was a devastating complication that can occur at any time [21]. The rate of late aneurysm rupture in the present study was comparable to that in previous studies. This complication must not be neglected, and close long-term follow-up after EVAR is required [7, 21–25].

Aortic events were defined as hospital death and re-intervention in the present study. The rate of freedom from aortic events was significantly higher in the OAR group than in the EVAR group in both the total cohort and propensity score-matched cohort. The re-intervention rate for OAR is reportedly 2.0–18%, and that of EVAR is 16–28% [5, 7, 22, 23, 26, 27], although the definition of re-intervention varies among studies. Our study found that the re-intervention rate of OAR was 7.2%, and that of EVAR was 15% (p < 0.0001). When focusing on only re-intervention requiring surgical repair, the re-intervention rate of OAR was 3.1%, and that of EVAR was 15%. Half of all re-interventions in the OAR group were simply for ileus. In the EVAR group, all patients with re-intervention required surgical repair. Scallan et al. reported an excellent late re-intervention rate of 2.0% [16]. However, those authors distinguished early re-intervention from late re-intervention. Furthermore, although ileus was defined as requiring re-intervention in our study, it was not included in some previous studies. Therefore, when reporting the re-intervention rate of OAR, the definition of re-intervention matters. The favorable re-intervention rates observed following OAR may be attributed to certain physical characteristics within the Japanese population, such as the low BMI, or even the surgical strategy utilized by replacing the abdominal aorta from just below the renal arteries.

Conversely, our re-intervention rate for EVAR was consistent with that described in previous reports. In our EVAR group, 83 re-interventions were performed in 63 patients. The most common cause for re-intervention was Type II endoleak (32 re-interventions). Open conversion was required for 24 (28%) of these re-interventions. As expected, the IFU group had a significantly higher rate of freedom from re-intervention than the non-IFU group. However, we must highlight the fact that the rate of freedom from re-intervention in the IFU group declined over time, indicating the need for close long-term follow-up, even in the IFU group.

Until recently, we had performed rapid proximal aortic clamping under laparotomy in patients with ruptured AAA. We were unable to use the operating room specialized for emergent EVAR. Furthermore, we had to order stent grafts for each case lacked a stock for emergent cases. Given the independent risk factors for aortic events in the OAR group and the elevating hospital mortality in this group as well, there is some room to reconsider this strategy for ruptured AAA. In unstable patients with ruptured AAA, rapid proximal aortic clamping is crucial. Recently, endovascular balloon endoclamping has been performed under local anesthesia just prior to the induction of general anesthesia in order to improve the outcome of ruptured AAA. From January 2020 to April 2021, we treated seven patients with ruptured AAA using the balloon endoclamp strategy; among these 7 patients, no hospital deaths have yet been observed. However, to decrease the rate of aortic events in the EVAR group, we need to decrease the occurrence of endoleak, especially type II. Our institution has been utilizing recently developed management strategies to this end, including preemptive embolization of not only the IMA but also the lumbar arteries. For the moment, no significant improvement with this approach has been demonstrated. However, further investigations are needed to investigate whether or not this method may improve the outcome of EVAR.

We must reiterate the following important findings from this study: the increased rate of aortic events, including rupture and open conversion, in the EVAR group; the increased re-intervention rate after EVAR even in the IFU group; and the lack of improvement in the re-intervention rate after EVAR even in the late term. Considering these issues, OAR remains our first-line strategy, although it is obvious that some patients do benefit from EVAR.

Several limitations associated with the present study warrant mention. First, this was a nonrandomized, single-center, retrospective study. Second, surgeons with vastly different levels of experience were involved in this study, and preemptive embolization was not performed successfully for all patients. There was a learning curve for the embolization technique. Third, we also found difficulty in defining aortic events. For example, retrograde ejaculation and erectile dysfunction were not included as aortic events. Fourth, the propensity score-matched analysis may itself be a limitation. To eliminate potential selection bias, propensity score matching was introduced. However, for example, “malignancy” and “frailty” were not included as perioperative variables for propensity matching. In addition, in patients with OAR, infrarenal and suprarenal aortic clamping were included. While suprarenal clamping is associated with increased morbidity and mortality compared to infrarenal clamping, some papers report that the clamping level is associated with the outcomes of OAR [28–30]. Finally, we used six different devices in this study and were unable to evaluate the individual features of each device. Furthermore, technological innovations have improved the performance of these devices, and newer devices might also be expected to provide better results than older ones. Further studies concerning each device are thus required.

## Conclusions

The OAR group had a significantly better overall survival than the EVAR group. The freedom from AAA-related death in both the OAR and EVAR groups was favorable. However, two late deaths due to rupture of AAA were identified in the EVAR group, while there were no AAA-related related deaths in the OAR group. The rate of freedom from aortic events, including hospital mortality and re-intervention, was significantly lower in the EVAR group than in the OAR group. Close long-term follow-up is needed even in patients with a suitable anatomy for EVAR. Considering these outcomes, our current approach is an OAR-first strategy based on individual patient characteristics.
